# Bioluminescence Measurement of Time-Dependent Dynamic Changes of CYP-Mediated Cytotoxicity in CYP-Expressing Luminescent HepG2 Cells

**DOI:** 10.3390/ijms22062843

**Published:** 2021-03-11

**Authors:** Satoru Iwado, Satoshi Abe, Mitsuo Oshimura, Yasuhiro Kazuki, Yoshihiro Nakajima

**Affiliations:** 1 Chromosome Engineering Research Center, Tottori University, 86 Nishi-cho, Yonago 683-8503, Tottori, Japan; iwado@live.jp (S.I.); sabe@trans-chromo.com (S.A.); moshimura@trans-chromo.com (M.O.); 2 Division of Genome and Cellular Functions, Department of Molecular and Cellular Biology, School of Life Science, Faculty of Medicine, Tottori University, 86 Nishi-cho, Yonago 683-8503, Tottori, Japan; 3 Health Research Institute, National Institute of Advanced Industrial Science and Technology (AIST), 2217-14 Hayashi-cho, Takamatsu 761-0395, Kagawa, Japan

**Keywords:** CYP, hepatotoxicity, HepG2 cells, luciferase, mouse artificial chromosome vector, real-time bioluminescence measurement

## Abstract

We sought to develop a cell-based cytotoxicity assay using human hepatocytes, which reflect the effects of drug-metabolizing enzymes on cytotoxicity. In this study, we generated luminescent human hepatoblastoma HepG2 cells using the mouse artificial chromosome vector, in which click beetle luciferase alone or luciferase and major drug-metabolizing enzymes (CYP2C9, CYP2C19, CYP2D6, and CYP3A4) are expressed, and monitored the time-dependent changes of CYP-mediated cytotoxicity expression by bioluminescence measurement. Real-time bioluminescence measurement revealed that compared with CYP-non-expressing cells, the luminescence intensity of CYP-expressing cells rapidly decreased when the cells were treated with low concentrations of aflatoxin B1 or primaquine, which exhibits cytotoxicity in the presence of CYP3A4 or CYP2D6, respectively. Using kinetics data obtained by the real-time bioluminescence measurement, we estimated the time-dependent changes of 50% inhibitory concentration (IC_50_) values in the aflatoxin B1- and primaquine-treated cell lines. The first IC_50_ value was detected much earlier and at a lower concentration in primaquine-treated CYP-expressing HepG2 cells than in primaquine-treated CYP-non-expressing cells, and the decrease of IC_50_ values was much faster in the former than the latter. Thus, we successfully monitored time- and concentration-dependent dynamic changes of CYP-mediated cytotoxicity expression in CYP-expressing luminescent HepG2 cells by means of real-time bioluminescence measurement.

## 1. Introduction

The human liver is the main site for drug metabolism and toxicities caused by the generation of reactive metabolites [1]. Primary human hepatocytes are considered the gold standard for assessing drug metabolism and hepatotoxicity in vitro because hepatic metabolism including CYP expression is highly conserved [2]. However, several problems have emerged in the use of primary human hepatocytes, including differences between lots, short life span, and high cost, which have limited screening for compounds by cell-based assay. Alternative models have been developed to overcome these problems [3,4,5].

Human hepatoblastoma cell line HepG2 is widely used to analyze cellular functions because of its homogeneity, unlimited life span, and low cost. However, the utility of HepG2 cells for assessing drug metabolism and toxicity is limited by the low expression of drug-metabolizing enzymes [6,7]. To solve this problem, we previously generated HepG2 cells using the mouse artificial chromosome (MAC) vector, in which major drug-metabolizing CYP enzymes (CYP2C9, CYP2C19, CYP2D6, and CYP3A4) and CYP oxidoreductase (POR) are constitutively expressed under the control of CAG promoter (hereinafter referred to as CYPs-HepG2 cells) [8]. These CYPs account for more than 65% of CYP enzymes in the human liver [9]. We demonstrated that aflatoxin B1 and sterigmatocystin, whose toxicities are mediated by CYP, showed cytotoxicity to CYPs-HepG2 cells but not wild-type HepG2 cells. Thus, it is considered that CYPs-HepG2 cells are a useful tool to evaluate CYP-mediated cytotoxicity of compounds [8].

Fluorescence imaging using a fluorescent dye or a genetically encoded fluorescent protein has contributed immensely to the advancement of a wide variety of biological studies including toxicological research, and has emerged as a powerful tool to image an extensive array of samples ranging from single molecules to whole organisms [10,11]. However, because a fluorescent probe requires exogenous illumination to emit light, long-term and quantitative fluorescence imaging must be very carefully performed for the following reasons: (i) the fluorescent probe is bleached by repetitive illumination; (ii) repetitive exogenous illumination causes phototoxic damage to cells; and (iii) exogenous illumination perturbs the physiology of light-sensitive tissues.

The bioluminescent reporter gene, luciferase, is broadly employed to quantitatively monitor cellular functions [12,13,14]. Luciferase is widely used for the conventional evaluation of cytotoxicity, where a decrease of bioluminescence intensity accompanied by an increase of cytotoxicity is used as an index [15,16,17,18,19,20,21]. Among available luciferases, beetle luciferases have the advantage of precisely monitoring cytotoxicity, namely, it is possible to measure non-destructively the luminescence of luciferase-expressing cells because D-luciferin (benzothiazole), a bioluminescent substrate for beetle luciferases, is very stable in culture medium and easily penetrates cells [22,23,24]. Furthermore, beetle luciferases enable tracking of dynamic changes of target cellular events longitudinally by means of real-time bioluminescence measurement [13,25,26]. We therefore considered that the dynamics of cytotoxicity expression can be simply and precisely analyzed by applying the real-time bioluminescence measurement method.

In this study, we established CYP-expressing luminescent HepG2 cells by introducing Brazilian click beetle luciferase (Emerald Luc; ELuc) [27] into previously generated CYPs-HepG2 cells, and succeeded in monitoring time- and concentration-dependent dynamic changes of CYP-mediated cytotoxicity by real-time bioluminescence measurement.

## 2. Results

### 2.1. Generation of CYP-Expressing Luminescent HepG2 Cells

**Figure 1:**
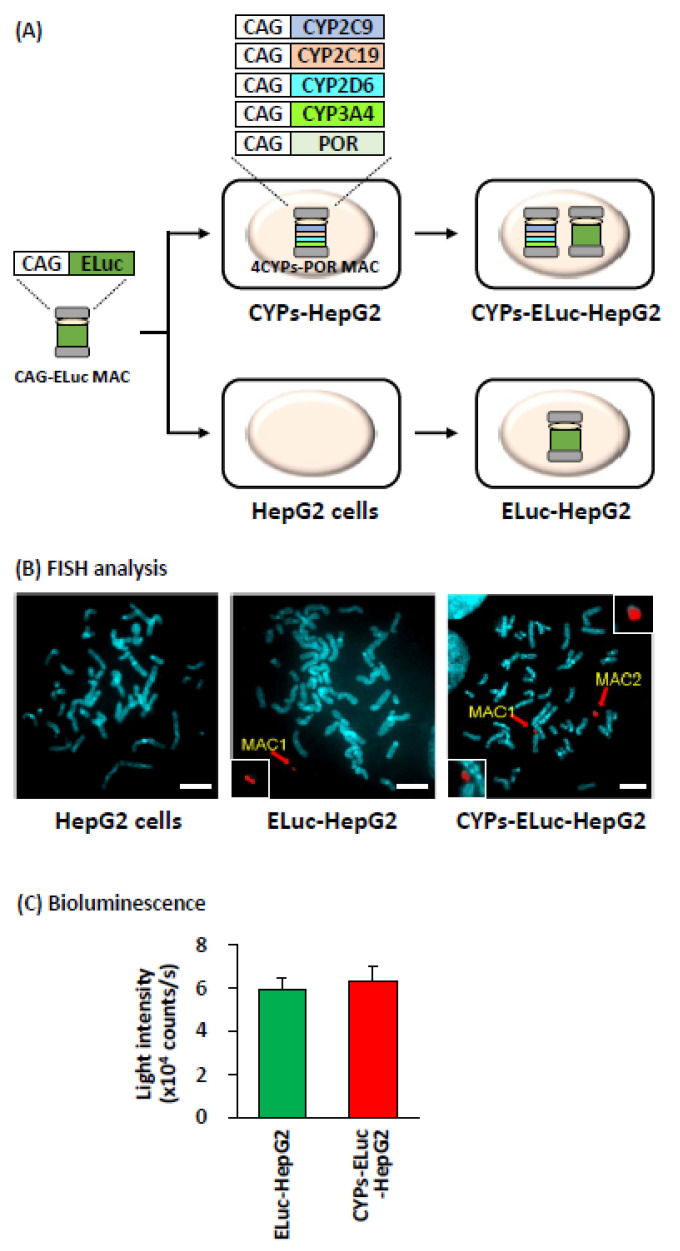
Generation of CYPs-ELuc-HepG2 and ELuc-HepG2 cells. (**A**) Schematic diagram for generating CYPs-ELuc-HepG2 and ELuc-HepG2 cells. CAG indicates CAG promoter. (**B**) FISH analysis of metaphase chromosome spreads from wild-type HepG2 cells (left panel), ELuc-HepG2 cells (middle panel), and CYPs-ELuc-HepG2 cells (right panel). Red arrows: MAC. Red signals: rhodamine-mouse Cot-1 DNA. Scale bar: 10 μm. The insets show enlarged images of each MAC. (**C**) Bioluminescence intensity of ELuc-HepG2 and CYPs-ELuc-HepG2 cells. One day after seeding, bioluminescence intensity was measured non-destructively for 5 s. Error bars indicate standard deviations (*n* = 3).

Figure 1A shows a scheme for preparing CYP-expressing luminescent HepG2 cells. In this study, we chose Brazilian click beetle luciferase Emerald Luc (ELuc) as the reporter gene because its bioluminescence intensity is much higher than that of other beetle luciferases [27]. To introduce ELuc gene into HepG2 cells, we used the MAC vector because transgene expression can be stably maintained during long-term culture [28,29], enabling stable cell-based cytotoxicity evaluation.

Microcells harboring the CAG-ELuc MAC vector, in which ELuc gene was connected to CAG promoter and inserted into a specific site of the MAC vector, were isolated from Chinese hamster ovary (CHO) cells harboring the CAG-ELuc MAC vector. The MAC vector was introduced by the measles virus envelope protein-mediated microcell-mediated chromosome transfer (MV-MMCT) method into CYPs-HepG2 cells established in a previous study [8], in which four major drug-metabolizing CYP enzymes (CYP2C9, CYP2C19, CY2D6, and CYP3A4) and CYP oxidoreductase (POR) were constitutively expressed under the control of CAG promoter, generating CYP-expressing luminescent HepG2 cells (hereinafter referred to as CYPs-ELuc-HepG2 cells). In parallel, the CAG-ELuc MAC vector was also introduced into wild-type HepG2 cells in the same way and the established cells were used for reference as CYP-non-expressing cells (hereinafter referred to as ELuc-HepG2 cells). Introduction of the CAG-ELuc MAC vector into wild-type and CYPs-HepG2 cells was confirmed by fluorescence in situ hybridization (FISH) analysis (Figure 1B) and genomic PCR (Supplementary Figure S1A). Non-destructive bioluminescence measurement revealed strong bioluminescence of both cell lines with almost the same intensity (Figure 1C). Finally, we also confirmed the remarkable activities of the four CYPs in CYPs-ELuc-HepG2 cells (Supplementary Figure S1B), as reported in a previous study [8].

### 2.2. Comparison of Sensitivity of ELuc-HepG2 and CYPs-ELuc HepG2 Cells to CYP-Independent Toxicants 

**Figure 2:**
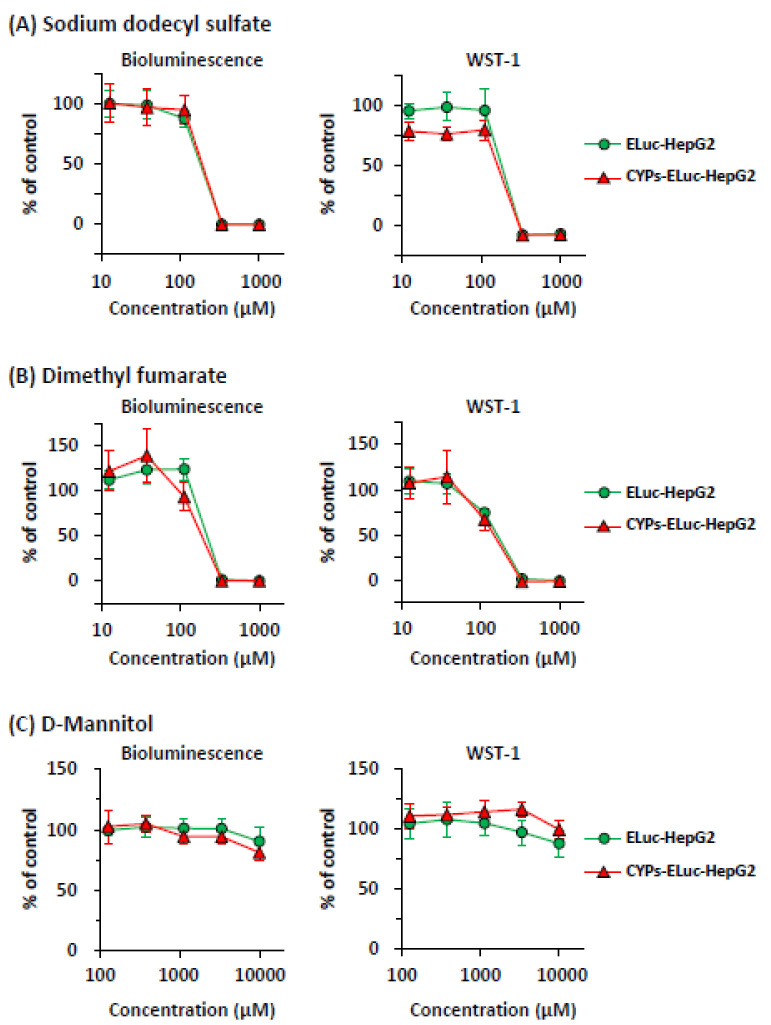
Effects of SDS, dimethyl fumarate, and D-mannitol on bioluminescence intensity and viability measured by WST-1 assay in ELuc-HepG2 and CYPs-ELuc-HepG2 cells. After the cells were treated with SDS (**A**), dimethyl fumarate (**B**), and D-mannitol (**C**) for 3 days, bioluminescence intensity was measured non-destructively for 5 s (left panels). Then, the WST-1 assay was conducted using the same cells (right panels). Data are expressed as percentage of vehicle control cells (set at 100%) and shown as means ± standard deviations (*n* = 3).

Next, to verify the sensitivity of the two cell lines to toxicants whose toxicity does not depend on CYP metabolism, we conducted non-destructive bioluminescence measurement and water-soluble tetrazolium-1 (WST-1) assay in parallel (Figure 2). After ELuc-HepG2 and CYPs-ELuc-HepG2 cells were treated with toxicant, the cells were incubated in the presence of D-luciferin, a bioluminescent substrate for ELuc, for three days and bioluminescence intensity was measured non-destructively thereafter, as reported previously [20,21]. Then, the WST-1 assay was performed using the same cells.

As shown in Figure 2A (left panel), the two cell lines treated with non-selective toxicant sodium dodecyl sulfate (SDS) showed very similar concentration-response curves in the bioluminescence measurement. The same tendency was noted in the WST-1 assay (right panel). Similar results were obtained when dimethyl fumarate, whose toxicity does not depend on CYP metabolism [30], was used to treat ELuc-HepG2 and CYPs-ELuc-HepG2 cells (Figure 2B). On the other hand, no remarkable changes were observed in the bioluminescence measurement and the WST-1 assay when non-toxicant D-mannitol was used to treat the two cell lines (Figure 2C). These results demonstrate that the sensitivities of ELuc-HepG2 and CYPs-ELuc-HepG2 cells to a toxicant whose toxicity does not depend on CYP metabolism are very similar, and that the decrease of bioluminescence intensity of cells well correlates with cytotoxicity assessed by the conventional cell viability assay, as reported previously by us [19,20] and others [16,31].

### 2.3. CYP-Mediated Cytotoxicity in CYPs-ELuc-HepG2 Cells

**Figure 3:**
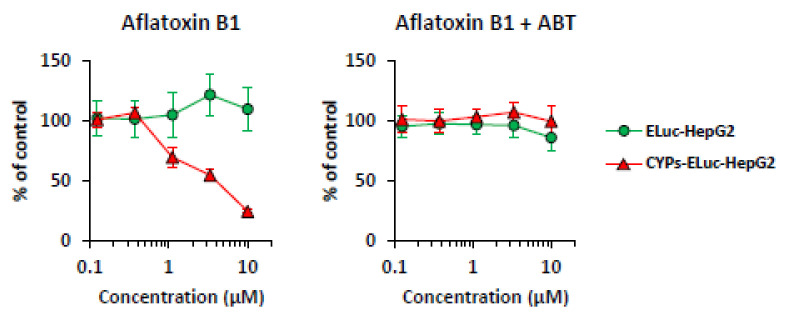
Effects of aflatoxin B1 and ABT on bioluminescence intensity in ELuc-HepG2 and CYPs-ELuc-HepG2 cells. ELuc-HepG2 (circles) and CYPs-ELuc-HepG2 cells (triangles) were treated with aflatoxin B1 in the absence (left panel) or presence (right panel) of 1 mM ABT. After 3 days, bioluminescence intensity was measured non-destructively for 5 s. Data are expressed as percentage of vehicle control cells (set at 100%) and shown as means ± standard deviations (*n* = 3).

We previously demonstrated that aflatoxin B1, whose toxicity mainly depends on CYP3A4 metabolism [32,33], clearly showed cytotoxicity to CYPs-HepG2 cells compared with wild-type HepG2 cells [8]. To verify whether the CYP-mediated cytotoxicity in CYPs-ELuc-HepG2 cells can be monitored by bioluminescence measurement, ELuc-HepG2 and CYPs-ELuc-HepG2 cells were treated with aflatoxin B1 and then subjected to non-destructive bioluminescence measurement. As shown in Figure 3 (left panel), whereas bioluminescence intensity did not decrease in ELuc-HepG2 cells treated with 10 μM aflatoxin B1 (circles), a concentration-dependent decrease of the bioluminescence intensity was observed in CYPs-ELuc-HepG2 cells (triangles), as previously observed in a neutral red uptake assay [8]. Importantly, the aflatoxin B1 concentration-dependent decrease of bioluminescence intensity in CYPs-ELuc-HepG2 cells was completely diminished by co-treatment with non-selective CYP inhibitor 1-aminobenzotriazole (ABT, 1 mM) (right panel, triangles). We noted that the bioluminescence intensities of both cell lines were not affected by the treatment with 1 mM ABT alone (Supplementary Figure S2). These results clearly demonstrate that CYP-mediated cytotoxicity is exhibited in CYPs-ELuc-HepG2 cells generated in this study, as in a previous report [8], and that the CYP-mediated cytotoxicity expression can be assessed by non-destructive bioluminescence measurement.

### 2.4. Stability of CYP-Mediated Cytotoxicity in CYPs-ELuc-HepG2 Cells during Long-Term Culture

In the cell-based cytotoxicity assay, it is important to obtain reproducible assay results during repetitive culture of cells. Therefore, we continuously cultured CYPs-ELuc-HepG2 cells in the absence of an antibiotic until population doubling level (PDL)-50, and the effects of aflatoxin B1 (a CYP-dependent toxicant), dimethyl fumarate (a CYP-independent toxicant), and D-mannitol (a non-toxicant) on the cultured CYPs-ELuc-HepG2 cells were evaluated by non-destructive bioluminescence measurement. 

**Figure 4:**
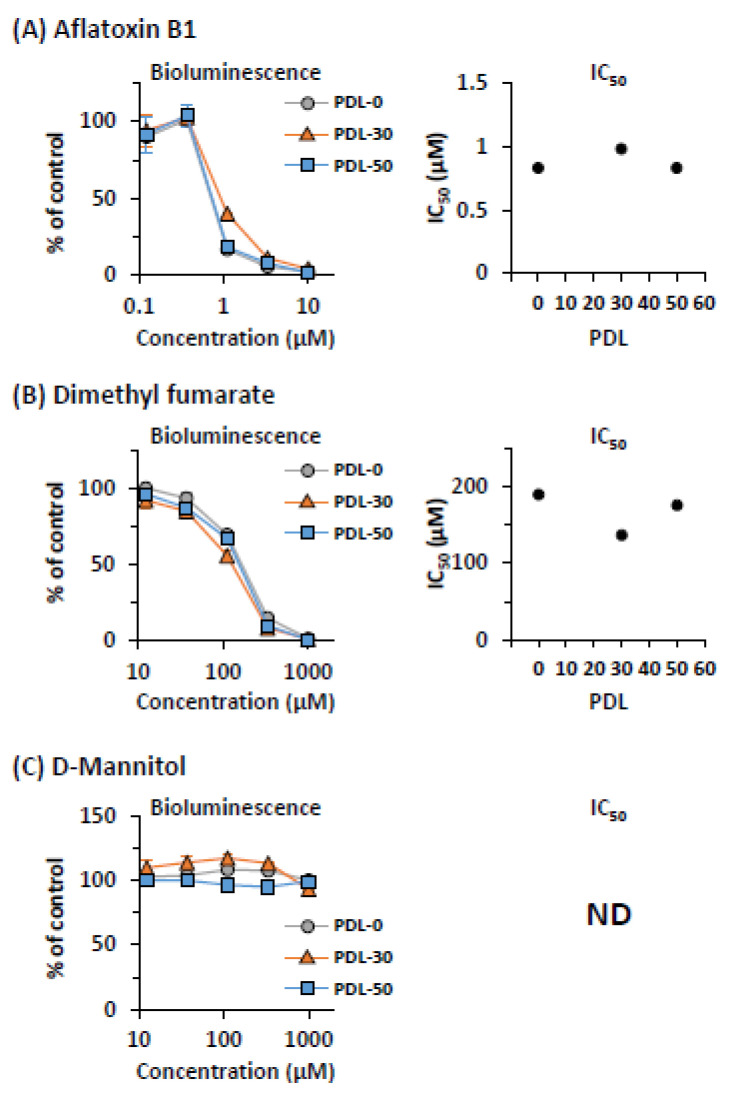
Stability of effects of aflatoxin, dimethyl fumarate, and D-mannitol on bioluminescence intensity in CYPs-ELuc-HepG2 cells during long-term culture. CYPs-ELuc-HepG2 cells at PDL-0, −30, and −50 were treated with aflatoxin B1 (**A**), dimethyl fumarate (**B**), and D-mannitol (**C**). Bioluminescence intensity was measured non-destructively for 5 s (left panels). Data are expressed as percentage of vehicle control cells (set at 100%) and shown as means ± standard deviations (*n* = 3). IC_50_ value at each PDL was calculated from the concentration-response curve obtained by bioluminescence measurement (right panel).

As shown in Figure 4A (left panel), the concentration-dependent decrease of bioluminescence intensity by aflatoxin B1 treatment at PDL-0 was observed (circles). Interestingly, identical concentration-response curves were obtained at PDL-30 and PDL-50. The 50% inhibitory concentration (IC_50_) values were almost unchanged even at PDL-50 (right panel). Similar results were obtained for dimethyl fumarate-treated CYPs-ELuc-HepG2 cells (Figure 4B). On the other hand, no significant changes of bioluminescence intensities were observed by the treatment with D-mannitol at every PDL (Figure 4C). We noted that the basal bioluminescence intensities of CYPs-ELuc-HepG2 cells were almost unchanged during long-term culture, and that stable assay results at PDL-30 and PDL-50, as shown in Figure 4, were also confirmed in CYPs-ELuc-HepG2 cells that were cultured in the presence of an antibiotic (400 μg/mL G418, data not shown). These results clearly demonstrate that CYP-mediated cytotoxicity can be assessed stably and longitudinally by bioluminescence measurement using CYP-expressing luminescent HepG2 cells. This may be due to the stable expression of the four CYPs and POR and the maintenance of CYP activities in CYPs-ELuc-HepG2 cells, as was previously observed in CYPs-HepG2 cells [8].

### 2.5. Real-Time Bioluminescence Measurement of CYP-Mediated Cytotoxicity in CYPs-ELuc-HepG2 Cells

**Figure 5:**
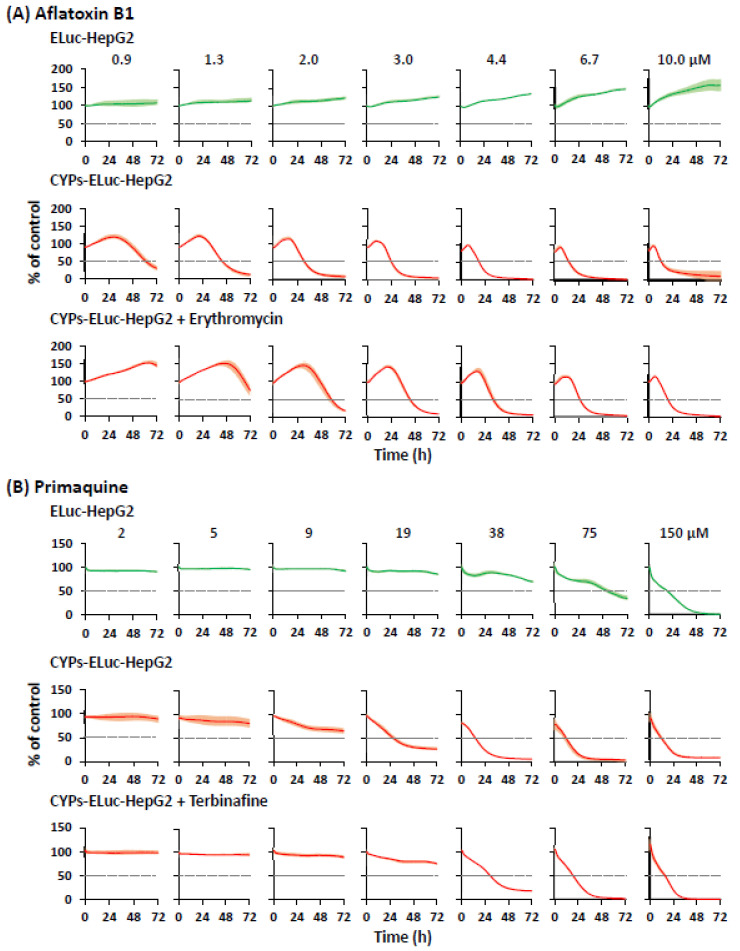
Real-time bioluminescence measurement of aflatoxin B1- or primaquine-treated ELuc-HepG2 and CYPs-ELuc-HepG2 cells. ELuc-HepG2 cells (upper panels) and CYPs-ELuc-HepG2 (middle panels) were treated with aflatoxin B1 (**A**) or primaquine (**B**), or co-treated with aflatoxin B1 plus CYP3A4 specific inhibitor erythromycin (12 μM) (**A**, bottom panels) or primaquine plus CYP2D6 specific inhibitor terbinafine (1 μM) (**B**, bottom panels). Bioluminescence was measured in real time for 5 s at 15-min intervals for 3 days. Data are expressed as percentage of vehicle control cells (set at 100%) at each time point and shown as means ± standard deviations (*n* = 3).

One advantage of bioluminescence measurement using beetle luciferases is that the bioluminescence of cells can be measured non-destructively in real time, enabling longitudinal tracking of cellular events [13,25,26]. Thus, as our next step, we examined whether the dynamics of CYP-mediated cytotoxicity expression in CYPs-ELuc-HepG2 cells could be tracked by real-time bioluminescence measurement (Figure 5). ELuc-HepG2 or CYPs-ELuc-HepG2 cells were seeded in 96-well plates, and bioluminescence was measured at 15-min intervals for 72 h in real time. In the aflatoxin B1-treated ELuc-HepG2 cells, no decrease of bioluminescence intensity was observed even at 10 μM, as in Figure 3 (Figure 5A, upper panels). In contrast, in CYPs-ELuc-HepG2 cells, time- and concentration-dependent decreases of bioluminescence intensity were observed (Figure 5A, middle panels), but these decreases were partially diminished by CYP3A4 specific inhibitor erythromycin (Figure 5A, lower panels and Supplementary Figure S3A) [34,35]. Real-time bioluminescence measurement was also conducted to evaluate the effects of primaquine, which shows CYP2D6-mediated cytotoxicity [36,37]. Time- and concentration-dependent decreases of bioluminescence intensity in ELuc-HepG2 cells were observed at 75 and 150 μM (Figure 5B, upper panels). A much faster decrease of bioluminescence intensity at a lower concentration was observed in CYPs-ELuc-HepG2 cells (Figure 5B, middle panels) than in ELuc-HepG2 cells. The decrease of bioluminescence intensity was partially abolished by terbinafine (Figure 5B, lower panels and Supplementary Figure S3B), a CYP2D6 specific inhibitor [38,39]. It should be noted that the time- and concentration-dependent changes of bioluminescence intensity in ELuc-HepG2 and CYPs-ELuc-HepG2 cells were almost identical when the cells were treated with dimethyl fumarate (a CYP-independent toxicant) (Supplementary Figure S4), as shown in Figure 2. 

**Figure 6:**
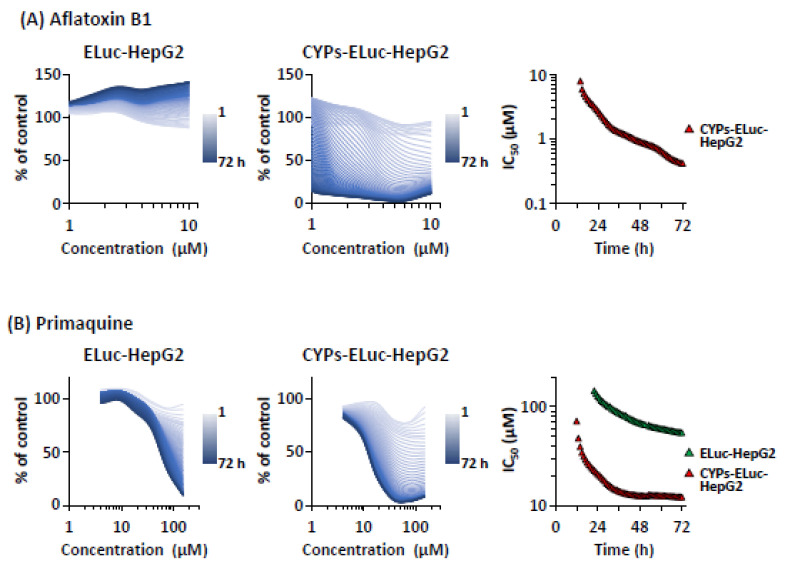
Concentration-response curves and time-dependent changes of IC_50_ values for aflatoxin B1- or primaquine-treated ELuc-HepG2 and CYPs-ELuc-HepG2 cells. Concentration-response curves at 1-h intervals were constructed using real-time bioluminescence measurement data shown in Figure 5 for aflatoxin B1- (**A**) or primaquine- (**B**) treated ELuc-HepG2 cells (left panels) and CYPs-ELuc-HepG2 cells (middle panels). Data are expressed as percentage of vehicle control cells (set at 100%) at each time point. IC_50_ values were calculated from the concentration-response curves at each time point.

Next, to precisely analyze the dynamics of CYP-mediated cytotoxicity expression, we prepared concentration-response curves at 1-h intervals (a total of 71 curves) using the real-time bioluminescence measurement results shown in Figure 5. Whereas 50% inhibitory concentration (IC_50_) was not observed in aflatoxin B1-treated ELuc-HepG2 cells (Figure 6A, left panel), the first IC_50_ value was noted at 14 h (7.9 μM) in aflatoxin B1-treated CYPs-ELuc-HepG2 cells (Figure 6A, middle and right panels), and this value decreased with measurement time. In primaquine-treated ELuc-HepG2 cells, the first IC_50_ value was confirmed at 22 h (145 μM), and this IC_50_ value slowly decreased until the end of measurement (Figure 6B, left and right panels). On the other hand, in primaquine-treated CYPs-ELuc-HepG2 cells, the first IC_50_ value appeared at a much earlier time and a lower concentration (12 h and 71 μM, respectively), and this IC_50_ value decreased rapidly until around 40 h, becoming constant thereafter (Figure 6B, middle and right panels). Thus, we were able to successfully monitor the dynamics of CYP-mediated cytotoxicity expression by real-time bioluminescence measurement using CYP-expressing luminescent HepG2 cells.

## 3. Discussion

In this study, we generated luminescent HepG2 cells expressing major CYP enzymes involved in drug metabolism using the MAC vector, and monitored the dynamics of CYP-mediated cytotoxicity expression by bioluminescence measurement.

First, to examine the appropriateness of cytotoxicity assessment by bioluminescence measurement, we conducted non-destructive bioluminescence measurement and the WST-1 assay in parallel using CYP-non-expressing luminescent HepG2 cells (ELuc-HepG2 cells) and CYP-expressing luminescent HepG2 cells (CYPs-ELuc-HepG2 cells). We confirmed that the decrease of bioluminescence intensity well correlated with the increase of cytotoxicity, as reported previously [19,20,21]. Furthermore, we obtained similar concentration-response curves for SDS- (non-selective toxicant) or dimethyl fumarate- (CYP-independent toxicant) treated ELuc-HepG2 and CYPs-ELuc-HepG2 cells, respectively (Figure 2), indicating that the sensitivity of the two cell lines to CYP-independent cytotoxicity is almost the same. On the other hand, the bioluminescence intensity of CYPs-ELuc-HepG2 cells was remarkably decreased by treatment with aflatoxin B1 and primaquine, which exhibit cytotoxicity when metabolized by CYP3A4 and CYP2D6, respectively. The decreases of the bioluminescence intensities were partially inhibited by CYP inhibitors (Figure 3 and Figure 5, Supplementary Figure S3). The results indicate that the CYP-expressing luminescent HepG2 cells generated in this study show CYP-mediated sensitivity to toxicant, and that measurement of bioluminescence intensity of the cells can accurately evaluate their sensitivity to toxicant.

In this study, we applied real-time bioluminescence measurement to track the dynamics of CYP-mediated cytotoxicity expression (Figure 5 and Figure 6, Supplementary Figures S3 and S4). The time- and concentration-dependent changes of bioluminescence intensity of dimethyl fumarate-treated ELuc-HepG2 and CYPs-ELuc-HepG2 cells showed almost the same kinetics (Supplementary Figure S4). On the other hand, when the two cell lines were treated with aflatoxin B1 and primaquine, CYPs-ELuc-HepG2 cells showed a much faster decrease of bioluminescence intensity than ELuc-HepG2 cells (Figure 5). 

We expressed the dynamics of cytotoxicity expression in both cell lines with the most well-used toxicity parameter, IC_50_. Whereas IC_50_ values were not observed in aflatoxin B1-treated ELuc-HepG2 cells under the experimental conditions, prompt appearance and continuous decrease of IC_50_ values were observed in aflatoxin B1-treated CYPs-ELuc-HepG2 cells. Compared with the IC_50_ values and their gradual decrease in primaquine-treated ELuc-HepG2 cells, the IC_50_ values appeared much earlier and at a lower concentration in primaquine-treated CYPs-ELuc-HepG2 cells (Figure 6). Thus, it was possible to track the dynamics of CYP-mediated cytotoxicity expression by real-time bioluminescence measurement using CYP-expressing luminescent HepG2 cells. In this study, we used aflatoxin B1 and primaquine to demonstrate CYP-mediated dynamic changes of bioluminescence intensity. However, it is reasonable to assume that CYPs-ELuc-HepG2 cells can also be used to evaluate the dynamics of CYP2C9- and CYP2C19-mediated cytotoxicity expression because the cells also expressed these drug-metabolizing enzymes (Supplementary Figure S1B).

In the present study, we chose ELuc to monitor cytotoxicity because it produces stronger bioluminescence than other beetle luciferases including firefly luciferase [27]. It is known that D-luciferin, a bioluminescent substrate for ELuc, is very stable in culture medium and not oxidized by other components in the medium. Therefore, D-luciferin has an extremely low background compared with other kinds of bioluminescent substrates [13,26]. In addition, D-luciferin easily permeates cells. Furthermore, as external illumination is not required in the bioluminescence reaction, the possibility of phototoxicity to cells and bleaching of test compound could be excluded. These characteristic properties of D-luciferin and luciferase are preferred to robustly perform real-time bioluminescence measurement, as was done in this study (Figure 5 and Figure 6, Supplementary Figures S3 and S4).

To express ELuc gene in HepG2 cells, we used the CAG promoter, which is an artificial promoter that is composed of cytomegalovirus immediate early enhancer, chicken β-actin promoter, and rabbit β-globin intron II [40]. By the combined use of ELuc and CAG promoter, we succeeded in generating very bright and stable HepG2 cell lines. The estimated bioluminescence intensities of ELuc-HepG2 and CYPs-ELuc-HepG2 cells were approximately 1 × 10^8^ photons per second, when bioluminescence was measured using a luminometer whose absolute optical responsivity was calibrated, as reported previously [41,42]. In the cytotoxicity evaluation using luciferase, the basal light intensity of cells is a very important property. Because luminescence of the cells decreases with increasing cytotoxicity, bright cells have a wider dynamic range than dark cells, enabling much more accurate cytotoxicity evaluation.

In this study, we used the MAC vector to generate CYP-expressing luminescent HepG2 cells. The cells were generated by introducing the CAG-ELuc MAC vector into previously established CYPs-HepG2 cells harboring the 4CYPs-POR MAC vector [8], in which four CYP genes and POR gene were inserted into the MAC vector. We previously reported that the expression and activities of the four CYPs were sustained stably during long-term culture [8]. Similarly, in this study, we confirmed that dimethyl fumarate, a non-CYP substrate, as well as aflatoxin B1, which exhibits CYP3A4-mediated cytotoxicity, shows stable cytotoxicity to CYPs-ELuc-HepG2 cells until PDL-50 even in the absence of antibiotic (Figure 4). We also confirmed that cytotoxicity assessed by bioluminescence measurement in aflatoxin B1-treated CYPs-ELuc-HepG2 cells at PDL-30 and PDL-50 was partially abolished by CYP3A4 specific inhibitor erythromycin (data not shown), as shown in Figure 5. These observations suggest that the expression and activities of transgenes (CYPs and ELuc), which are inserted into the MAC vector in CYPs-ELuc-HepG2 cells, are stably maintained, and that the translocation of the MAC vector into the chromosome of HepG2 cells does not occur even if two copies of the MAC vector coexist in a cell. Thus, it is reasonable to consider that the CYP-expressing luminescent HepG2 cells generated in this study are appropriate to stably and accurately evaluate cytotoxicity by bioluminescence measurement.

We have successfully monitored the dynamics of CYP-mediated cytotoxicity expression by combining chromosome engineering and bioluminescence technologies. Although we used the 96-well plate format, a more high-throughput format, such as 384- or 1536-well plates, could be used because the bioluminescence intensity of luminescent HepG2 cells is very strong. Through bioluminescence measurement using CYPs-ELuc-HepG2 cells alone or in combination with non-CYP-expressing ELuc-HepG2 cells, CYP-mediated cytotoxicity can be simply and accurately evaluated with a high-throughput format.

## 4. Materials and Methods

### 4.1. Chemicals

Primaquine and erythromycin were obtained from FUJIFILM Wako Pure Chemical Corporation (Tokyo, Japan). Aflatoxin B1 and dimethyl fumarate were obtained from Sigma–Aldrich (St. Louis, MO, USA). Terbinafine was obtained from Tokyo Chemical Industry (Tokyo, Japan). These compounds were dissolved in dimethyl sulfoxide (DMSO, Sigma–Aldrich). The final concentration of DMSO in culture medium was less than 0.1%. SDS and D-mannitol were purchased from Nacalai Tesque (Kyoto, Japan) and dissolved in distilled water.

### 4.2. Cell Culture

Hypoxanthine phosphoribosyl transferase-deficient CHO) (JCRB0218, Japanese Collection of Research Bioresources, Osaka, Japan) derived cells were maintained in Ham’s F-12 nutrient mixture (FUJIFILM Wako Pure Chemical Corporation) supplemented with 10% fetal bovine serum (FBS; HyClone Laboratories, Logan, UT, USA). CHO cells harboring the CAG-ELuc MAC vector were generated by a specific insertion of the pCAG-ELuc plasmid [19] into the R4 attP site of the MAC vector, as reported previously [21]. HepG2-derived cells (RCB1886, RIKEN BRC, Ibaraki, Japan) were maintained in Dulbecco’s modified Eagle’s medium (DMEM; FUJIFILM Wako Pure Chemical Corporation) supplemented with 10% FBS (HyClone Laboratories), 4 mM L-glutamine, non-essential amino acids (Gibco-BRL, Grand Island, NY, USA), 1 mM pyruvate, 100 units/mL penicillin G, and 100 μg/mL streptomycin (FUJIFILM Wako Pure Chemical Corporation). 

### 4.3. Microcell-Mediated Chromosome Transfer (MMCT)

Measles virus (MV) envelope protein-mediated MMCT (MV-MMCT) was performed as described previously [43,44]. Briefly, CHO cells harboring the CAG-ELuc MAC vector and expressing MV-hemagglutinin (MV-H) and MV-fusion (MV-F) proteins were used as donor microcell hybrids. We transiently expressed MV-H and MV-F in CHO cells harboring the CAG-ELuc MAC vector by transient transfection of their expression vectors. On the next day, the cells were transferred into flasks, and after an overnight incubation, micronucleus formation in CHO cells was induced by a 72-h treatment with 0.1 μg/mL colcemid (Thermo Fisher Scientific, Waltham, MA, USA). The cells were centrifuged with cytochalasin B (Sigma–Aldrich) to isolate microcells. The collected microcells were cocultured with wild-type HepG2 cells or CYPs-HepG2 cells harboring the 4CYPs-POR MAC vector to induce the fusion of micronuclei with the recipient cells. To select HepG2 cells harboring the CAG-ELuc MAC vector, cells were cultured in media containing 2 µg/mL blasticidin S (FUJIFILM Wako Pure Chemical Corporation). 

### 4.4. FISH Analysis

FISH analysis was performed by using spreads of fixed chromosomes in either metaphase or interphase from each cell hybrid and a digoxigenin-labeled (Roche Diagnostics, Mannheim, Germany) mouse cot-1 DNA probe (Invitrogen, Carlsbad, CA, USA). The digoxigenin-labeled DNA was detected with anti-digoxigenin-rhodamine complex (Roche Diagnostics). Chromosomal DNA was counterstained with 4,6-diamidino-2-phenylindole (DAPI; Sigma–Aldrich). Metaphase images were captured digitally using a CoolCube1 charged-coupled device camera (MetaSystems GmbH, Altlussheim, Germany) coupled with an AxioImagerZ2 fluorescence microscope with a 63 ×1.4 NA oil objective and appropriate filter cubes (Carl Zeiss GmbH, Jena, Germany). Images were processed using ISIS software provided with the microscope (MetaSystems).

### 4.5. Genomic PCR

Genomic DNA was extracted from wild-type HepG2, ELuc-HepG2, and CYPs-ELuc-HepG2 cells using a genomic DNA extraction kit with DNase-free RNase (Gentra Systems, Minneapolis, MN, USA), according to the manufacturer’s instructions. Genomic PCR was performed using the following primer sets: ELuc-F (5′-CTCGAGGTCGACGGTATCGA-3′) and ELuc-R (5′-AATGTATCTTATCATGTCTG-3′) for amplification of ELuc gene.

### 4.6. Measurement of CYP Activities

Measurement of the activities of all four CYPs was conducted using the P450-Glo assay (Promega, Madison, WI, USA), according to the manufacturer’s instructions. Briefly, ELuc-HepG2 cells, CYPs-ELuc-HepG2 cells, and CHO cells harboring the 4CYPs-POR-MAC vector were seeded in 96-well plates at a density of 1 × 10^4^ cells/well. Then, 24 h later, the luminescent substrate included in the kit was added into the culture medium. After incubation for 1 h, the detection reagent was added, and the luminescence generated by the metabolic substrate in the culture medium was measured using an Infinite F500 plate reader (Tecan, Zurich, Switzerland). 

### 4.7. Non-Destructive Bioluminescence Measurement and WST-1 Assay

ELuc-HepG2 or CYPs-ELuc-HepG2 cells were seeded in 96-well black clear bottom plates (Nunc, Wiesbaden, Germany) at a density of 1 × 10^4^ cells per well. After an overnight incubation, the medium was replaced with DMEM without phenol red (Gibco-BRL) but with 10% FBS (HyClone Laboratories), 4 mM glutamine, non-essential amino acids (Gibco-BRL), 1 mM pyruvate (FUJIFILM Wako Pure Chemical Corporation), 25 mM HEPES/NaOH (pH 7.0, Sigma–Aldrich), and 300 μM D-luciferin potassium salt (Resem B.V., Lijnden, The Netherlands) with or without compound. After incubation for 3 days in a CO_2_ incubator, ELuc luminescence intensity in the cells was measured. The 96-well plate was set in a microplate-type luminometer (AB-2350 Phelios; ATTO, Tokyo, Japan) and luminescence was measured without disrupting the cells for 5 s. For the WST-1 assay, Premix WST-1 reagent (Takara Bio Inc., Shiga, Japan) was added to the remaining HepG2 cells and incubation was carried out for 1 to 2 h at 37 °C. Absorbance was measured at 450 nm (reference 620 nm) using the microplate reader Infinite 200 PRO (Tecan). 

### 4.8. Real-Time Bioluminescence Measurement

ELuc-HepG2 or CYPs-ELuc-HepG2 cells were seeded in 96-well black clear-bottom plates (Nunc) at a density of 1 × 10^4^ cells/well. After an overnight incubation, the medium was replaced with DMEM without phenol red (Gibco-BRL) supplemented with 10% FBS (HyClone Laboratories), 4 mM glutamine, non-essential amino acids (Gibco-BRL), 1 mM pyruvate (FUJIFILM Wako Pure Chemical Corporation), 25 mM HEPES/NaOH (pH 7.0, Sigma–Aldrich), and 300 μM D-luciferin potassium salt (Resem B.V.) with or without compound. Real-time bioluminescence measurement was carried out using a microplate-type luminometer (WSL-1565 Kronos HT, ATTO) for 5 s at 15-min intervals for 3 days. During bioluminescence measurement, the cells were maintained at 37 °C and 5% CO_2_ and under saturated humidity condition.

## Data Availability

Four supplemental data files are available as Supplementary Data (above).

## Supplementary Materials

The following are available online at https://www.mdpi.com/1422-0067/22/6/2843/s1, Figure S1: Characterization of ELuc-HepG2 and CYPs-ELuc-HepG2 cells by genomic PCR and CYP activity analyses, Figure S2: Bioluminescence intensity in the absence or presence of 1 mM ABT in ELuc-HepG2 and CYPs-ELuc-HepG2 cells, Figure S3: Real-time bioluminescence measurement of aflatoxin B1- or primaquine-treated CYPs-ELuc-HepG2 cells in the absence or presence of CYP inhibitor, Figure S4: Real-time bioluminescence measurement of dimethyl fumarate-treated ELuc-HepG2 and CYPs-ELuc-HepG2 cells.
