# Supplementary material for: Bioluminescence Measurement of Time-Dependent Dynamic Changes of CYP-Mediated Cytotoxicity in CYP-Expressing Luminescent HepG2 Cells
Source: Int J Mol Sci. 2021 Mar 11;22(6):2843. doi: 10.3390/ijms22062843 (PMC7999318; doi:10.3390/ijms22062843)
Supplement: Supplementary file 1 [file ijms-22-02843-s001.pdf]

(A) Genomic PCR

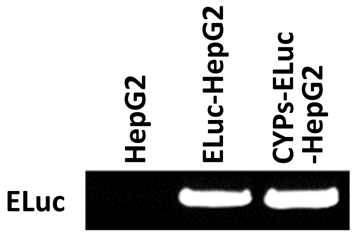

(B) CYP activities

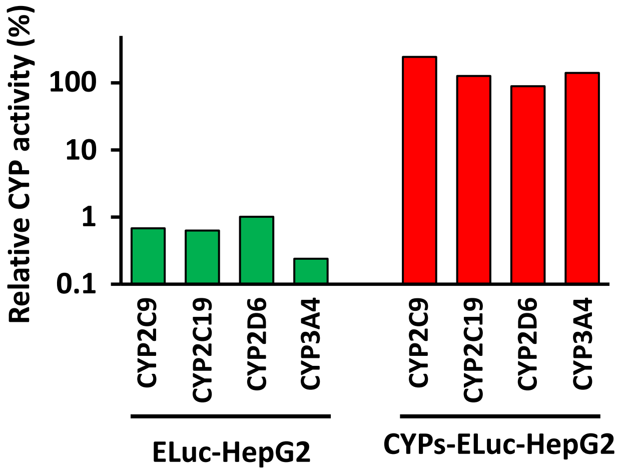

Supplementary figure S1. Characterization of ELuc-HepG2 and CYPs-ELuc-HepG2 cells by genomic PCR and CYP activity analyses. (A) Genomic PCR for amplification of ELuc cDNA in wild-type HepG2, ELuc-HepG2, and CYPs-ELuc-HepG2 cells. (B) CYP activities in ELuc-HepG2 and CYPs-ELuc-HepG2 cells. Activity is expressed as percentage of each CYP activity in parent CHO cells harboring the 4CYPs-POR MAC vector (set at 100%).

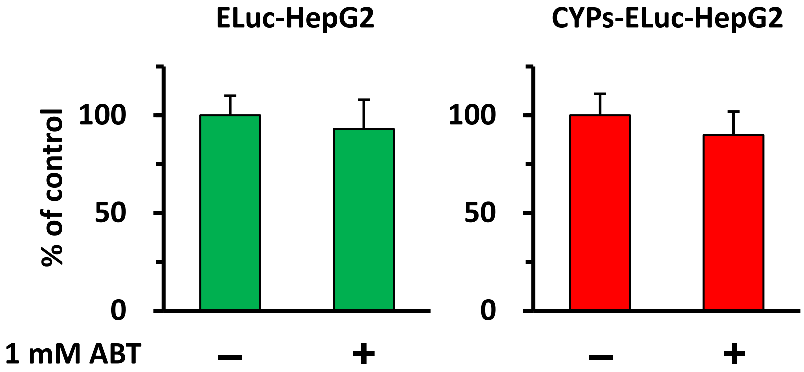

Supplementary figure S2. Bioluminescence intensity in the absence or presence of 1 mM ABT in ELuc-HepG2 and CYPs-ELuc-HepG2 cells. Cells were incubated for 3 days in the absence or presence of 1 mM ABT, and bioluminescence intensity was measured non-destructively. Data are expressed as percentage of vehicle control cells (set at 100%) and shown as means ± standard deviations (*n* = 3).

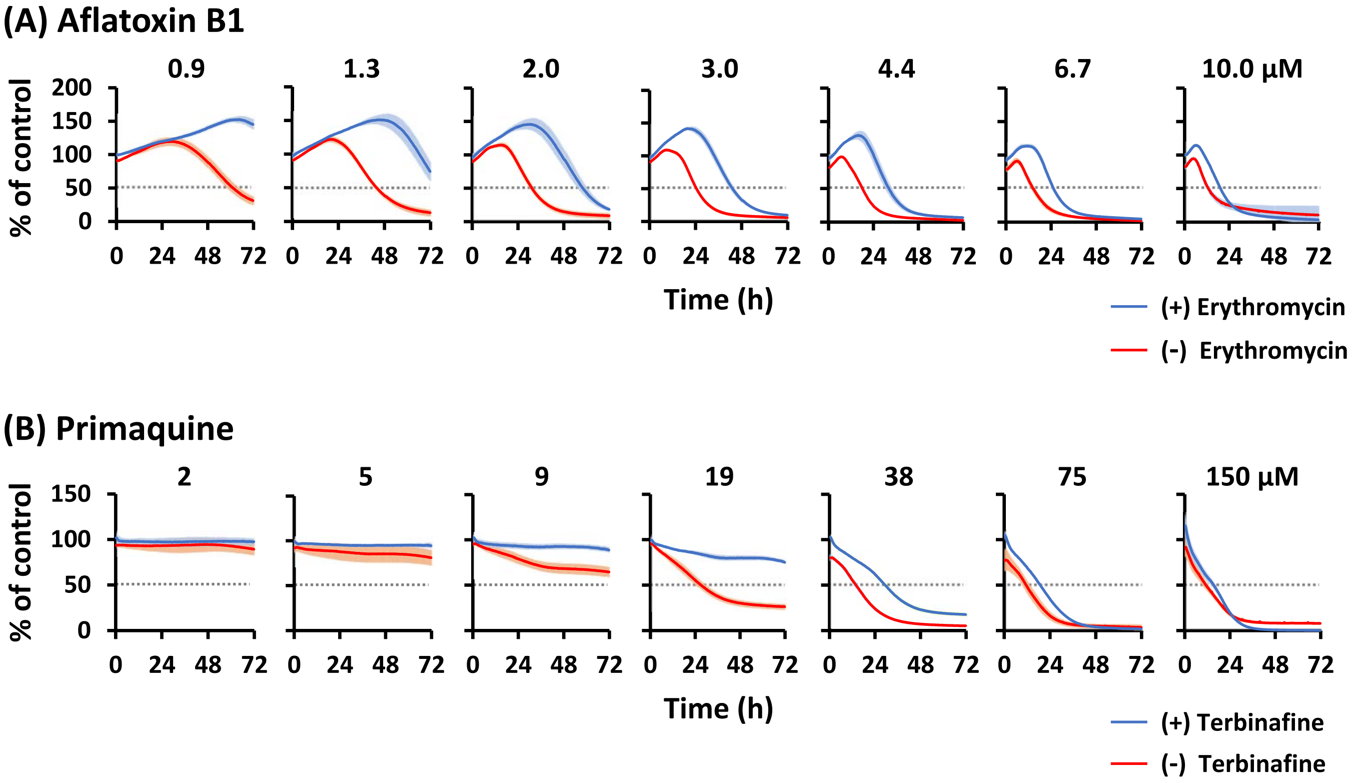

Supplementary figure S3. Real-time bioluminescence measurement of aflatoxin B1- or primaquine-treated CYPs-ELuc-HepG2 cells in the absence or presence of CYP inhibitor. Real-time bioluminescence measurement data for CYPs-ELuc-HepG2 cells shown in Figure 5 were merged. Red and blue lines indicate bioluminescence measurement data in the absence and presence of CYP inhibitor, respectively. Data are expressed as percentage of vehicle control cells (set at 100%) at each time point and shown as means ± standard deviations (*n* = 3).

Dimethyl fumarate

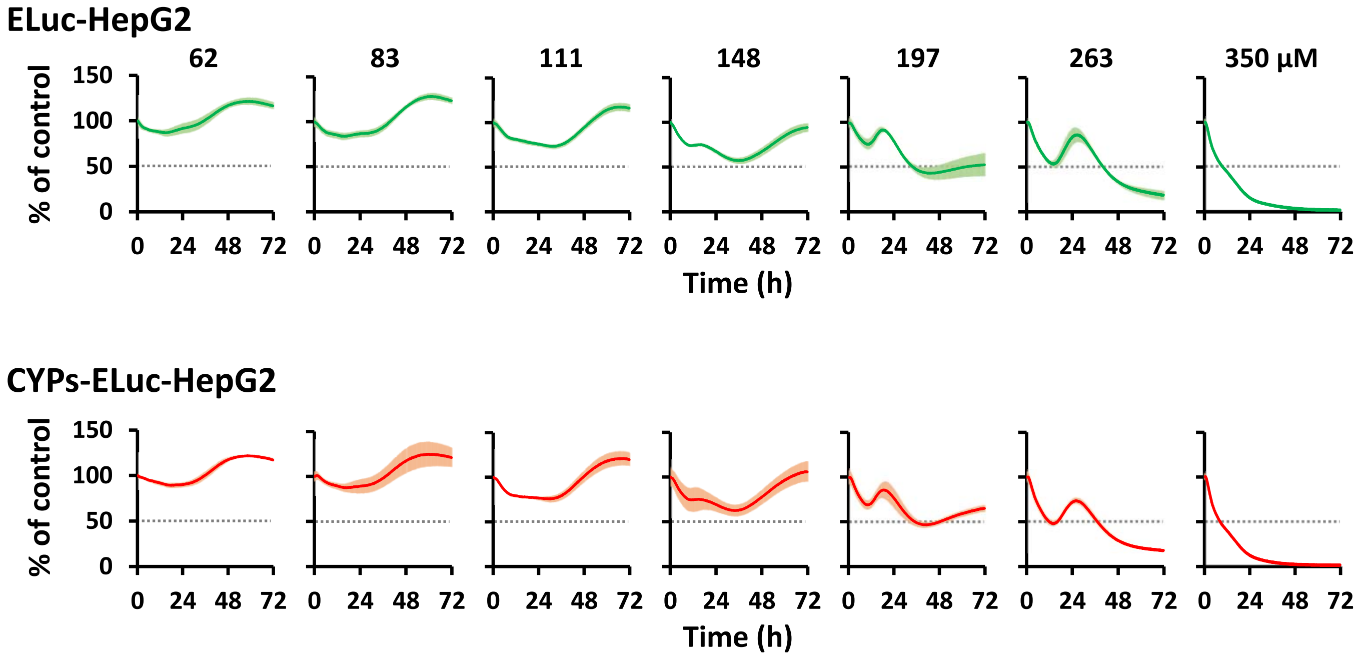

Supplementary figure S4. Real-time bioluminescence measurement of dimethyl fumarate-treated ELuc-HepG2 and CYPs-ELuc-HepG2 cells. ELuc-HepG2 cells (upper panel) or CYPs-ELuc-HepG2 cells were treated with dimethyl fumarate, and bioluminescence was measured in real time for 5 s at 15-min intervals for 3 days. Data are expressed as percentage of vehicle control cells (set at 100%) at each time point and shown as means ± standard deviations (n = 3).
